# Delivery of a patient-friendly functioning report to improve patient-centeredness of dialysis care: a pilot study

**DOI:** 10.1186/s12913-019-4733-6

**Published:** 2019-11-27

**Authors:** Laura C. Plantinga, Brian Jones, Jeremy Johnson, Amelia Lambeth, Janice P. Lea, Leigh Nadel, Ann E. Vandenberg, C. Barrett Bowling

**Affiliations:** 1 Department of Medicine, Emory University, Atlanta, Georgia USA; 2 Department of Epidemiology, Rollins School of Public Health, Emory University, Atlanta, Georgia USA; 3 Interactive Media Technology Center, Georgia Institute of Technology, Atlanta, Georgia USA; 4 Durham Veterans Affairs Geriatric Research Education and Clinical Center, Durham Veterans Affairs Medical Center, Durham, North Carolina USA; 5 Department of Medicine, Duke University, Durham, North Carolina USA

**Keywords:** Physical functioning, Patient-centered care, Patient-provider communication, Hemodialysis

## Abstract

**Background:**

Provider recognition of level of functioning may be suboptimal in the dialysis setting, and this lack of recognition may lead to less patient-centered care. We aimed to assess whether delivery of an app-based, individualized functioning report would improve patients’ perceptions of patient-centeredness of care.

**Methods:**

In this pre-post pilot study at three outpatient dialysis facilities in metropolitan Atlanta, an individualized functioning report—including information on physical performance, perceived physical functioning, and community mobility—was delivered to patients receiving hemodialysis (*n* = 43) and their providers. Qualitative and quantitative approaches were used to gather patient and provider feedback to develop and assess the report and app. Paired *t* test was used to test for differences in patient perception of patient-centeredness of care (PPPC) scores (range, 1 = most patient-centered to 4 = least patient-centered) 1 month after report delivery.

**Results:**

Delivery of the reports to both patients and providers was not associated with a subsequent change in patients’ perceptions of patient-centeredness of their care (follow-up vs. baseline PPPC scores of 2.35 vs. 2.36; *P* > 0.9). However, patients and providers generally saw the potential of the report to improve the patient-centeredness of care and reacted positively to the individualized reports delivered in the pilot. Patients also reported willingness to undergo future assessments. However, while two-thirds of surveyed providers reported always or sometimes discussing the reports they received, most (98%) participating patients reported that no one on the dialysis care team had discussed the report with them within 1 month.

**Conclusions:**

Potential lack of fidelity to the intervention precludes definitive conclusions about effects of the report on patient-centeredness of care. The disconnect between patients’ and providers’ perceptions of discussions of the report warrants future study. However, this study introduces a novel, individualized, multi-domain functional report that is easily implemented in the setting of hemodialysis. Our pilot study provides guidance for improving its use both clinically and in future pragmatic research studies, both within and beyond the dialysis population.

## Background

Providing patient-centered care in the setting of dialysis is challenging and may require a significant shift in practice [1, 2]. For example, patient-provider discussion of physical functioning is outside the scope of usual dialysis care. However, such discussions could lead to better recognition of function-related issues and to more patient-centered communication, which fosters the patient-provider relationship, responds to patient emotions, exchanges information, and enables shared decision-making and disease- and treatment-related patient behavior [3].

Decreased physical functioning is common in dialysis [4–12]; is a strong predictor of increased mortality, morbidity, and healthcare utilization [10, 13–17]; and is itself an important patient-centered outcome [18, 19] among patients receiving dialysis. However, recognition of poor physical functioning by dialysis providers is suboptimal [20]. Despite this, there are no interventions to improve recognition or facilitate discussion of physical functioning in this population. Thus, we aimed to design an app that can be used to collect data on physical functioning and generate an individualized report of patients’ relative multi-domain functional status. We then conducted a pilot study to measure patient perceptions of patient-centeredness before and after delivery of the app-based report to patients receiving hemodialysis and their providers.

## Methods

### Report and app development

#### Development of initial report

The initial report was generated by the [INstant Functional Outcomes Report for Meaningful Encounters in Dialysis (INFORMED)] team. The report included: (*i*) physical performance [Short Physical Performance Battery (SPPB), total score scale 0–12 (subscales of balance, gait speed, and chair stands scale 0–4); higher scores = better performance] [21]; (*ii*) patient-reported perceived physical functioning [Physical Functioning (PF) score (scale 0–100; higher scores = higher perceived functioning) included in the Kidney Disease Quality of Life] [22]; (*iii*) patient-reported activity limitations [basic activities of daily living (BADLs) [23] and instrumental activities of daily living (IADLs)] [24]; (*iv*) patient-reported history of falls from items assessing falls and their causes in the previous year and fear of falling during daily tasks [Falls Efficacy Scale (scale 0–100; higher scores = greater fear of falling; score ≥ 70 = fear of falling)] [25]; and (*v*) patient-reported community mobility [UAB Study of Aging Life-Space Assessment (LSA) instrument, with modifications excluding travel to the dialysis center; scale 0–120, higher scores = greater community mobility] [26]. This initial report (Additional file 1: Figure S1, *left*), representing a hypothetical patient on hemodialysis, was reviewed by our Advisory Group and a literacy expert before it was used in focus groups.

#### Focus groups

Methods for the INFORMED focus groups have been described [27]. Briefly, focus group participants were purposively recruited at three Emory-affiliated dialysis centers for four 90-min focus groups (held in 3/17; two with patients receiving hemodialysis, one with physicians, and one with nurses, social workers, and dietitians). Participants were provided a copy of the hypothetical report, and discussions about the report were recorded and transcribed verbatim. Feedback from all four groups was used to make final changes to the report prior to app development.

#### App development

The development team used an agile software development project management process, in which the research team described the desired features of the app. User feedback on the app was sought and captured in an iterative development process, in which current features were improved and new features added, until the delivery of the final working app. The app’s primary functions were to support researchers in administration of the various patient assessments, generate and print the resulting physical functioning reports to a wireless printer, and capture that data for later use by researchers. The app guided the researcher through administration of each assessment, including recording of responses by the researcher and integrated timers and verbal script prompts for the SPPB. When assessments were completed, the app generated a physical functioning report that could be printed and exported.

### Pilot study

#### Recruitment

All patients receiving in-center hemodialysis on the second and third shifts of two dialysis facilities were targeted for recruitment via phone call (2/18–7/18; *n* = 246). Of the *N* = 105 patients reached via phone or self-referral, *N* = 52 agreed to participate. Of these, *N* = 43 completed the baseline study visit (Additional file 1: Figure S2).

#### Patient study visits

In the baseline visit, measurement of physical performance via the SPPB was performed before a scheduled dialysis session to minimize the risk of falls due to post-dialysis hypotension; Mondays and Tuesdays were excluded due to potential effects of a 3-day interdialytic period. Self-reported information on functioning was collected via survey while the patient dialyzed on the same day. The entire assessment via app took approximately 30 min. All functioning data were entered into the INFORMED app. Patients were also surveyed during the baseline visit about the patient-centeredness of dialysis care using a modified version of the Patient Perception of Patient-centeredness of Care (PPPC) [28] survey (Additional file 1: Table S1). In the 1-month follow-up visit, patients were surveyed while dialyzing using the same PPPC instrument and a survey about the utility of the report (Additional file 1: Table S2), and data were entered into REDCap [29].

#### Report delivery

Individualized paper reports were delivered to patient participants immediately after measurements were completed. Reports were delivered via secure email to the patient’s providers (nephrologist, social worker, dietitian) within 2 days of the baseline study visit. Reports were also uploaded to the dialysis facilities’ electronic health record (EHR); access instructions were included in the initial emails sent to providers with the reports (Additional file 1: Box S1). Monthly reminders to review the reports were sent to all providers who had received reports that month, in the week prior to interdisciplinary rounds (Additional file 1: Box S1).

#### Provider survey

When all baseline patient visits were complete, all providers who had received ≥1 report (*n* = 17) were asked to complete a survey regarding the utility of the survey (Additional file 1: Table S3). The online survey was distributed via email, and responses and data were collected and managed via REDCap [29].

#### Statistical analysis

Patient characteristics, including PPPC scores, functioning data, and demographic and clinical data (obtained from the patients’ dialysis records via linkage), were summarized. Baseline and follow-up PPPC scores were calculated as the mean of all 14 items; subscores were calculated similarly [28] (Additional file 1: Table S1). Paired *t* tests were used to compare follow-up vs. baseline PPPC scores. Responses to quantitative items on the patient and provider utility surveys (Additional file 1: Tables S2 and S3) were reported as percentages. All analyses were performed with Stata v 14.2 (College Station, TX). Supplementary figures to visualize intersecting sets of impairments were created using apps in R [30, 31].

## Results

### Functioning report development

Patients (mean age, 53) in the focus groups were primarily (88%) black and predominantly male (65%); in contrast, about half of providers in the focus groups (mean age, 49) were black (53%) and 65% of providers were female (Table 1). Generally, all stakeholder groups discussed the potential of the report to improve the patient-centeredness of care and, particularly, to track function longitudinally; patients and providers alike felt the utility of the report could be limited by the time and space restrictions of the dialysis facility and for patients whose function was either very low or high (Table 1).

**Table 1:** Characteristics of and general functioning report feedback from participants in the focus group, 3/17

	Patients Receiving Hemodialysis	Hemodialysis Providers
No. of patients	17	17
No. of focus groups^*^	2	2
Participant Characteristics		
Mean age, years	53	49
% male	65%	24%
% black	88%	53%
Mean years on dialysis/treating kidney patients	8 years	12 years
Report Feedback		
Perceived uses of the report:		
Facilitation of individualized/patient-centered care	“[I]t’s a gauge for each individual that has issues in their own way to address with a doctor.” —Patient group 1 “I think, if this is done more with dialysis patients a lot of things that transcends like weight gain, weight losses, and all that stuff can be controlled better. And you get a better understanding with the doctor and the patient on what they could do in order to kind of bring that into focus, you know, more readily with the patient.” —Patient group 1	“If I got a report like this … for example, this patient can easily feed themselves, but going grocery shopping and preparing their own food is not likely to happen. So this patient could benefit from a Meals-on-Wheels type program.” —Non-physician group “It’s good to build a rapport with your patients. You know, just talking to them about their status, functioning status, and offering support. So I think that’s a good way to … kind of build trust with them as well.” —Non-physician group
Potential longitudinal use	“I would take this whole chart and try to make it better.” —Patient group 2	“I can see where you could use the tool serially and someone is either getting better or worse.” —Physician group
Perceived limitations of the report:		
Limitations in the dialysis facility setting	“I don’t think the dialysis clinic would be the setting for something like this, I think it would be somewhere where you schedule an appointment at the convenience of the patient’s schedule or when the patient feels like they’re up to [it].” —Patient group 1	“Some of them have transportation [issues], they have to leave right away, they don’t have time to sit with you. Some prefer … more confidentiality, so it just depends, I think.” —Non-physician group
Limitations when functioning is very high or low	“Well it is OK for people that’s physically able to do it, but people like me [in a wheelchair] that’s not physically able, I would score 0 on every one of them.” —Patient group 1	“Obviously if you have amputations, like a lot of people do, or if you can’t walk or get up, you can’t do any of this, you’d get a zero. But you may have some functional capacity; if you’re in a wheelchair you might be able to wheel yourself around.” —Physician group

^*^For providers, focus groups were split by discipline: physicians and physician extenders vs. nurses, social workers, and dietitians

**Fig. 1:**
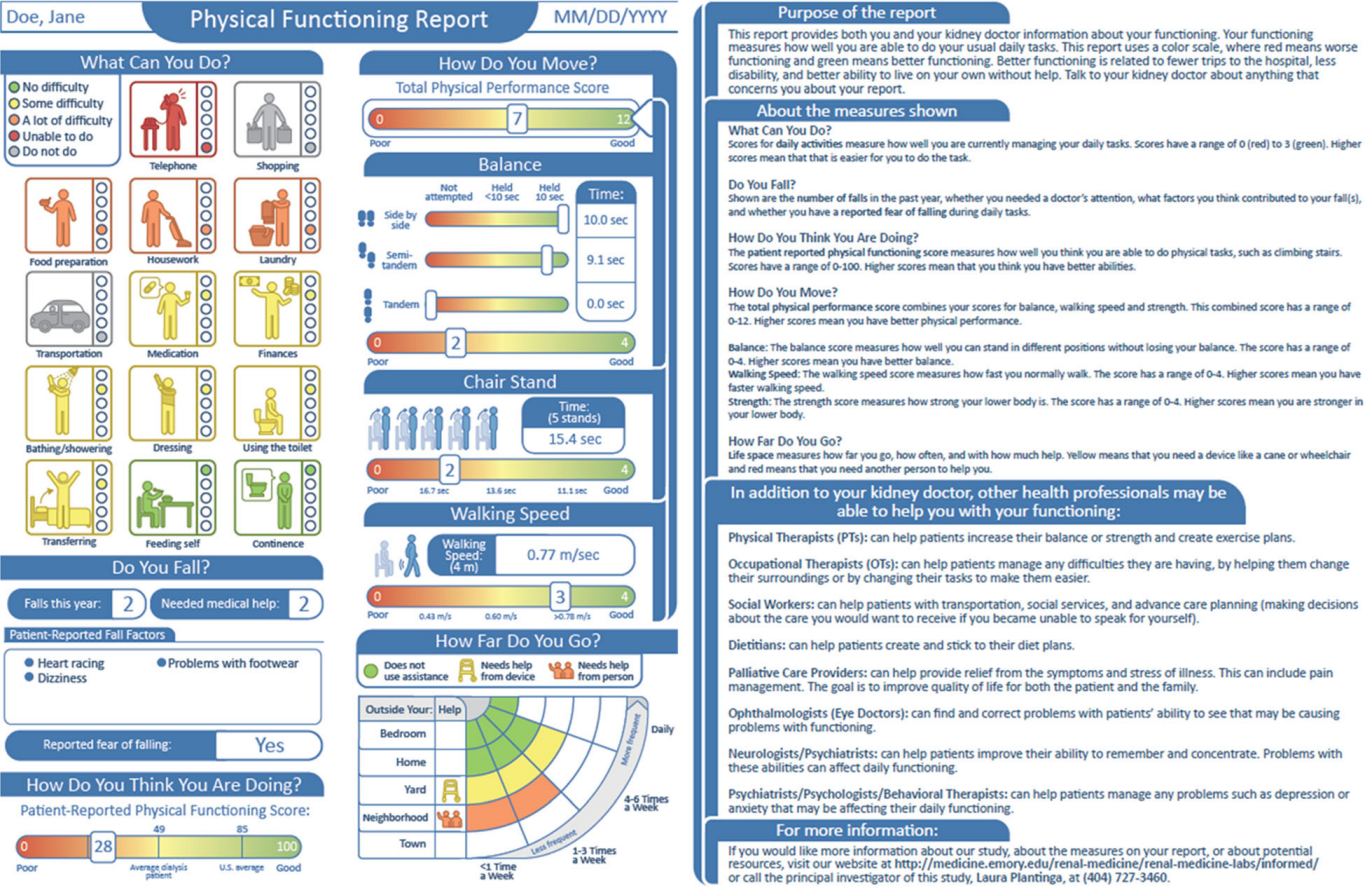
Final physical functioning report incorporating focus group feedback and delivered to patients and providers in the pilot study

Based on feedback from all stakeholder groups, we made several changes to the initial report (Additional file 1: Figure S1). Figure 1 shows the final report delivered to patient participants and providers in the pilot study. We also made changes to the pilot study protocol based on focus group feedback, including: (*i*) in-person training presentations at physician meetings and recorded webinars available on our study website [32]; (*ii*) delivery of reports to non-physician as well as physician providers; and (*iii*) delivery of electronic versions of the report to providers, both by email and uploaded to the EHR.

### Pilot study

#### Participants

Of the 105 participants we contacted, 52 were scheduled for a baseline visit, and 43 completed the first visit and received individualized reports [recruitment rate = 41.0%; retention rate = 82.7%; Additional file 1: Figure S2]. All participants who completed the first study visit completed the second visit [mean (SD) days between visits = 31.1 (6.1)]. On average, patients in the pilot were 57 years old; 49% were female and 92% were black (Table 2). Median scores for SPPB, perceived functioning, and LSA were 8, 65, and 54; however, we observed scores across the entire possible ranges. More than half of participants (56%) reported impairment in at least one IADL; 44% reported impairment in at least one BADL. More than one-third (35%) reported falling in the prior year (Table 2). Younger vs. older participants generally had higher functioning across all domains, although variability was high (Additional file 1: Table S4). While male participants had higher functioning across most domains than female participants, the differences were generally not statistically significant (Additional file 1: Table S4). Pairwise correlations between domains were weak to moderate, although perceived physical functioning was strongly associated with physical performance, BADLs/IADLs, and life-space scores (Additional file 1: Table S5). Visualization of functioning impairments suggested multiple patterns involving different domains of function among individuals in the cohort (Additional file 1: Figure S3).

**Table 2:** Characteristics and self-reported perceptions of patient-centeredness of care among 43 patients receiving hemodialysis who participated in the pilot, 2/18–8/18

Characteristic	Overall
Demographic and clinical characteristics	
Age, mean (SD)	56.5 (13.7)
Male, *n* (%)	22 (51.2%)
Black race, *n* (%)	36 (92.3%)
Median (IQR) years with ESRD	3.0 (1.4–6.2)
Primary attributed cause of ESRD, *n* (%)	
Diabetes	5 (11.6%)
Hypertension	33 (76.7%)
Other	5 (11.6%)
Functioning	
Median (IQR) SPPB score	8 (4–9)
Median (IQR) PF score	65 (40–80)
Impaired in any BADL, *n* (%)	19 (44.2%)
Impaired in any IADL, *n* (%)	24 (55.8%)
Fell in past year, *n* (%)	15 (34.9%)
Median (IQR) LSA score	54 (33–72)
Perceptions of patient-centeredness of dialysis care	
Mean (SD) PPPC total score, baseline	2.36 (0.74)
Mean (SD) PPPC total score, follow-up	2.35 (0.62)
P* for difference in score	*> 0.9*
Mean (SD) subscore, experience explored, baseline	2.17 (0.78)
Mean (SD) subscore, experience explored, follow-up	2.01 (0.74)
P* for difference in score	*0.2*
Mean (SD) subscore, common ground, baseline	2.51 (0.82)
Mean (SD) subscore, common ground, follow-up	2.58 (0.66)
P* for difference in score	*0.5*
Mean (SD) subscore, whole person, baseline	1.67 (0.71)
Mean (SD) subscore, whole person, follow-up	1.60 (0.69)
P* for difference in score	*0.5*

*N* = 43 for all variables listed, except race (*N* = 39) and years on dialysis (*N* = 41)

*BADL* basic activity of daily living, *ESRD* end-stage renal disease, *IADL* instrumental activity of daily living, *IQR* interquartile range, *LSA* Life-Space Assessment (scale, 0–120, *higher scores* greater community mobility), *PF* physical functioning subscale (scale 0–100, *higher scores* better perceived functioning), *PPPC* Patient Perception of Patient-Centeredness (scale, 1–4, *lower scores* more patient-centered), *SPPB* Short Physical Performance Battery (scale, 0–12, *higher scores* better performance)

*By paired *t* test. Note the statistical significance threshold, accounting for multiple testing overall and across subscales, would be 0.0125, by conservative (Bonferroni) correction

#### Reported patient-centeredness

At baseline, the mean total PPPC score was 2.36 (possible range 1–4, with lower scores indicating perceptions of more patient-centered care), while mean subscores indicating patients’ perceptions that illness experiences were explored, that common ground was found, and that the provider viewed the patient as whole person were 2.17, 2.51, and 1.67, respectively (Table 2). At 1-month follow-up, PPPC scores were not statistically significant different from baseline scores.

#### Patient-reported experiences with the report

At follow-up, 42/43 (97.7%) of patients reported that no one on the dialysis care team had discussed the report with them (Table 3). Among these 42 patients, 40.5% reported wanting to discuss the report with their provider, 11.9% brought up the report themselves, and 35.7% discussed it with someone outside of the dialysis care team. While none discussed with primary care or other non-dialysis providers or with spiritual advisors, many discussed it with family and friends (Table 3). More than half (54.8%) planned to discuss the report in a future visit with a provider (Table 3). All patients reported willingness to undergo future functioning assessment, at frequencies ranging from monthly/every 3 months (69.8%) to annually (Table 3).

**Table 3:** Patient experience with, and perceptions of the utility of, the functioning report, 3/18–8/18

Item	*n* (%)
No. (%) responded	43 (100%)
No. (%) reported that anyone on the dialysis care team discussed report with them	1 (2.3%)
Among 42 patients with complete follow-up:	
No. (%) wanted to discuss report with provider	17 (40.5%)
No. (%) brought up report with a dialysis provider themselves	5 (11.9%)
No. (%) discussed report with someone else not on dialysis care team	15 (35.7%)
Primary care provider or other non-dialysis provider	0 (0.0%)
Spouse	5 (11.9%)
Child	2 (4.8%)
Other relative	8 (19.1%)
Friend	2 (4.8%)
Spiritual or religious advisor	0 (0.0%)
No. (%) planned to discuss report in future visit with provider	23 (54.8%)
Among all 43 patients:	
No. (%) willing to undergo routine testing to receive reports:	
Monthly	15 (34.9%)
Every 3 months	15 (34.9%)
Every 6 months	8 (18.6%)
Annually	5 (11.6%)
Never	0 (0.0%)

#### Provider-reported experiences with the report

Of the 17 providers contacted, 12 (70.6% response rate) completed the survey (8/18–9/18). Provider type and responses are displayed in Table 4. Nearly half (41.7%) reported that no training was needed to increase comfort with discussing the report (Table 4). Most providers reported always (16.7%) or sometimes (50.0%) discussing the report with patients (Table 4). While most reported rarely or never providing recommendations based on the report, the most common recommendations listed by providers were patient-driven exercise programs and family consults. Most (87.5%) felt comfortable discussing the report. More than a third (37.5%) felt the report led to better communication with their patients (Table 4) because, for example, “results can be used to address ADLs” and “report disclosed information patient did not share with me or the care team.” For providers who suggested the functioning report did not improve communication, most stated that communication was already good with the patient(s). No providers felt they were unqualified to discuss the report, that it was not their role to discuss the report, or that the information was not important for patient care (Table 4).

**Table 4:** Hemodialysis provider experience with, and perceptions of the utility of, the functioning report, 8/18–9/18

Item	*n* (%)
No. (%) completing survey	12 (70.6%)
Type of hemodialysis provider	
Nephrologist	5 (41.7%)
Social Worker	4 (33.3%)
Dietitian	3 (25.0%)
No. (%) reporting receiving 0 reports:	2 (16.7%)
No. (%) reporting that report should be discussed by:	
Nephrologist	8 (66.7%)
Nurse	6 (50.0%
Social worker	6 (50.0%)
Dietitian	5 (41.7%)
Primary care provider	3 (25.0%)
Other	1 (8.3%)
No one	0 (0.0%)
No. (%) reporting discussing reports with patients:	
Always	2 (16.7%)
Sometimes	6 (50.0%)
Never	4 (33.3%)
No. (%) reporting training would improve their comfort discussing report:	
In-person training	3 (25.0%)
Web training	3 (25.0%)
Role modeling of provider-patient encounters	2 (16.7%)
Other	1 (8.3%)
No training needed	5 (41.7%)
Among 8 providers who *always* or *sometimes* discussed report with patients	
No. (%) reporting who brought up report:	
Patients brought it up more often	2 (25.0%)
Provider brought it up more often	4 (50.0%)
Patients/providers brought it up equally	2 (25.0%)
No. (%) reporting making recommendations after report discussion:	
Frequently/sometimes	3 (37.5%)
Rarely/never	5 (62.5%)
No. (%) reporting making recommendations for:	
Physical therapy	1 (12.5%)
Patient-driven exercise program	2 (25.0%)
Occupational therapy/home assessment	1 (12.5%)
Depression work-up	1 (12.5%)
Cognitive assessment	1 (12.5%)
New social service	0 (0.0%)
Family consult	2 (25.0%)
Other	0 (0.0%)
None	1 (12.5%)
No. (%) reporting they felt comfortable discussing the report with patients	7 (87.5%)
No. (%) reporting that report led to better communication with patient	3 (37.5%)
Among 10 providers who *sometimes* or *never* discussed report with patients	
No. (%) reporting reasons for not discussing report:	
It was not appropriate to discuss	1 (10.0%)
Provider felt unqualified to discuss	0 (0.0%)
Provider felt it was not their role to discuss	0 (0.0%)
There was never enough time	3 (30.0%)
Information was not important for patient care	0 (0.0%)
Information was not actionable	1 (10.0%)
Provider forgot about report	1 (10.0%)
Provider did not receive report	2 (20.0%)
Other	2 (20.0%)

## Discussion

In this pilot study of patients receiving hemodialysis and their providers, patient and provider reactions to the individualized functioning reports provided in the study were generally positive. However, while two-thirds of surveyed providers reported always or sometimes discussing the reports they received, most (98%) participating patients reported that no one on the dialysis care team had discussed the report with them at the time of survey. Delivery of the reports to both patients and providers was not associated with a subsequent change in patients’ perceptions of patient-centeredness of their care (follow-up vs. baseline PPPC scores of 2.35 vs. 2.36; *P* > 0.9).

Potential lack of fidelity to the intervention (i.e., lack of provider discussion of the report during encounters within 1 month of receipt) might explain our null results with respect to patient-centeredness of care. It is also possible that delivery of this individualized report would have had no effect on this score in the limited timeframe of our study. Additionally, the PPPC score [28] may not capture elements of patient-centeredness that are important specifically in hemodialysis care. Finally, the scores, while in the middle of the range of possible scores (1 = most patient-centered to 4 = least patient-centered), may already be maximized for in-center hemodialysis care as it is currently delivered in U.S. facilities, where provider visits are generally frequent but brief, and often unassociated with patient needs [33].

Interestingly, we observed a substantial mismatch between patients’ and providers’ responses regarding discussions of the report. If providers had discussions after the 1-month window, the lag between patient and provider surveys (up to 5 months for some patients) might partially explain this phenomenon. In fact, more than half (55%) of patients indicated that they planned to discuss the report in a future visit. There may also be social desirability bias in the providers’ responses regarding these discussions. However, patients and providers may also have different perceptions of “discussing the report”; e.g., if a physician reviewed a patient’s report and made a referral or brought up issues based on the information in the report, without mentioning the report, the physician (but not the patient) may perceive this as a discussion of the report. Similarly, the limited health literacy that is common in this population [34] may be an issue: in fact, patients with earlier-stage kidney disease often report that they do not understand what the nephrologist told them during visits and often do not even recall that a kidney disease diagnosis was ever discussed [35].

This disconnect between patients’ and providers’ perceptions regarding the discussion of the functioning report is similar to that in a study by Wachterman et al. [36], in which seriously ill (≥20% physician-assessed risk of dying in the next year) hemodialysis patients and their nephrologists were interviewed regarding their perceived prognosis. In most cases, the patients were more optimistic than their nephrologists, with 81% of patients estimating they had a ≥ 90% chance of survival in the next year, whereas nephrologists estimated this prognosis for only 25% of these patients. In fact, none of the patients in the study reported having ever received a prognosis from their nephrologists [36], suggesting that provision of information might align patients’ and providers’ estimates and thus allow them to be “on the same page” in discussion of goals of care. However, much like the Study to Understand Prognoses and Preferences for Outcomes and Risks of Treatments (SUPPORT) trial [37], which showed timely delivery of information on prognosis and patient preferences had no effect on end-of-life outcomes (including ratings of communication, knowledge of preferences for resuscitation, and high-burden utilization such as intensive care and mechanical ventilation) among seriously ill hospitalized patients, the results of our study suggest that information alone may not be enough. In fact, information is likely a necessary, but not sufficient, component of the type of open-ended discussion of functioning—and the eliciting of goals of care and shared decision-making in the context of functioning—that would improve the patient-centeredness of care, at least in the challenging environment in which dialysis care is delivered. Further, while dialysis facilities have a built-in multidisciplinary team of nurses, social workers, and dietitians, who could potentially have such discussions and implement needed support services, the care plans are generally driven by the nephrologists. The nephrologists’ action-oriented responses in our preliminary qualitative study, in which they expressed discomfort at receiving information that could not act upon and even expressed preferences for having a separate report to protect patients from receiving potentially negative information, suggest that willingness and/or ability to have such conversations is currently limited [27]. The lowest PPPC subscores we observed in this pilot were for “finding common ground,” further suggesting this may be the primary communication barrier to patient-centered care in this population. Future studies in this population may be improved with engagement of geriatricians or geriatric interprofessional teams as an alternative to engagement of nephrologists, who have been trained in a disease-oriented approach to care, which is further reinforced by required quality reporting that does not prioritize patient-centeredness [1, 2, 38].

Most patients wanted to discuss the reports with their providers, and they were willing to undergo testing to have the reports available longitudinally. Providers indicated that functioning information was useful clinically, that they felt comfortable with the functioning information, and that they felt it was primarily the job of dialysis providers to discuss the information conveyed by the report. Among those who discussed the report, more than a third noted that it led to better communication with their patients. Not surprisingly, dialysis providers noted that limited time was a barrier to the use of the report. Some providers did not remember receiving reports, and only a few tried to view the reports on the dialysis EHR, suggesting that the mode and timing of delivery of the reports may have been suboptimal for dialysis providers.

Functioning scores among these patients were low, consistent with prior studies [4–12], but varied considerably between individuals, with entire ranges of scores represented. This variability, combined with the limited overlap between domains of functioning within individuals, suggest a high degree of individuality in functioning among patients receiving hemodialysis. The strength of the multi-domain functioning report we delivered is that it captures this individuality and allows providers at-a-glance information on what interventions patients may need. For example, the providers in our pilot mentioned a wide variety of services (e.g., physical therapy, occupational therapy, depression work-up, and cognitive assessment) to which they referred based on the report. Importantly, the report also provides functioning information in a standardized way, avoiding the use of imperfect proxies of functioning (e.g., age or “eyeball test”) that may be used by clinicians in the absence of other information.

There are additional limitations to this study worth nothing. Recruitment among patients receiving hemodialysis was difficult, and the possibility of selection bias due to differential recruitment remains. It is also possible that the reminders to complete surveys may have incentivized lagging providers to view or have discussions about the report before completing the survey. We did not include nurses because they did not have assigned patients; however, as the providers noted, nurses are an integral part of the interdisciplinary care team and likely necessary for successful clinical implementation of these reports. We also did not include patient caregivers. In fact, family and friends—many of whom were likely caretakers—were the most commonly included individuals on the list of people with whom the patient had discussed the report. While not within the scope of this pilot study, having mandatory, scripted patient-provider discussions about the report may have mitigated concerns about the fidelity of the intervention, providing a better estimate of efficacy, rather than effectiveness. Additional measurements of patient-centeredness both before and after the delivery of the report might have captured changes more robustly, and the small sample size in this pilot precluded meaningful subgroup analyses. Finally, generalizability to populations of patients receiving hemodialysis that are older or have a different race/ethnicity or socioeconomic distribution from our metropolitan Atlanta population may be limited.

## Conclusions

Despite its limitations, this pilot study introduces a novel, individualized, multi-domain functional report that is easily implemented via app in the setting of hemodialysis. Likely lack of fidelity to intervention precludes conclusions about the effect of delivery of the report on patient-centeredness of patient-provider communication. However, the disconnect between patients’ and providers’ perceptions of discussions of the report highlights that provision of information may not be sufficient to support the open-ended discussions of functioning needed for eliciting of goals and shared decision-making that are critical to patient-centered care; this warrants future study. Furthermore, our findings will inform future studies to improve implementation of the app and report in the dialysis population and beyond.

## Supplementary information

**Additional file 1:** Supplementary tables and figures.

## Data Availability

De-identified versions of the datasets used in the current study are available from the corresponding author on reasonable request.

## Abbreviations

BADL: Basic Activity of Daily Living
IADL: Instrumental Activity of Daily Living
INFORMED: INstant Functional Outcomes Report for Meaningful Encounters in Dialysis
LSA: Life-Space Assessment
PF: Physical Functioning
PPPC`: Patient Perception of Patient-centeredness of Care
SPPB: Short Physical Performance Battery
SUPPORT: Study to Understand Prognoses and Preferences for Outcomes and Risks of Treatments
