# Supplementary material for: Delivery of a patient-friendly functioning report to improve patient-centeredness of dialysis care: a pilot study
Source: BMC Health Serv Res. 2019 Nov 27;19:891. doi: 10.1186/s12913-019-4733-6 (PMC6880368; doi:10.1186/s12913-019-4733-6)
Supplement: Supplementary file 1 — Additional file 1: Supplementary tables and figures. [file 12913_2019_4733_MOESM1_ESM.docx]

**Additional File 1: Supplementary Material**

**Title:** Delivery of a Patient-Friendly Functioning Report to Improve Patient-Centeredness of Dialysis Care: A Pilot Study

**Authors:** Laura C. Plantinga, Brian Jones, Jeremy Johnson, Amelia Lambeth, Janice P. Lea, Leigh Nadel, Ann E. Vandenberg, and C. Barrett Bowling

**Table S1.** Modified Patient Perceptions of Patient-Centeredness (PPPC) survey* used in pilot study of patients receiving hemodialysis.

| **Domain/item**** | **Possible responses and scores** |
| --- | --- |
| Patient perception that illness experience has been explored | |
| 1-To what extent was your physical functioning (in other words, how well you are getting around) discussed during the visit? | 1-Completely  2-Mostly  3-A little  4-Not at all |
| 2-Would you say that your doctor knows about your level of physical functioning? | 1-Yes  2-Probably  3-Unsure  4-No |
| 3-To what extent does the doctor understand the importance of your physical functioning? | 1-Completely  2-Mostly  3-A little  4-Not at all |
| 4-How well do you think your doctor understood you during the visit? | 1-Very well  2-Well  3-Somewhat  4-Not at all |
| Patient perception that patient and doctor found common ground | |
| 5-How satisfied were you with the discussion you had with your doctor during the visit? | 1-Very satisfied  2-Satisfied  3-Somewhat satisfied  4-Not satisfied |
| 6-To what extent did the doctor explain how you are doing? | 1-Completely  2-Mostly  3-A little  4-Not at all |
| 7-To what extent did you agree with the doctor's opinion about how you are doing? | 1-Completely  2-Mostly  3-A little  4-Not at all |
| 8-How much opportunity did you have to ask your questions? | 1-Very much  2-Fair amount  3-A little  4-Not at all |
| 9-To what extent did the doctor ask about your goals for treatment? | 1-Completely  2-Mostly  3-A little  4-Not at all |
| 10-To what extent did the doctor explain any new treatments, changes to your treatment, and/or referrals to other providers? | 1-Very well  2-Well  3-Somewhat  4-Not at all |
| 11-To what extent did the doctor explore how manageable any new treatment, change in treatment, and/or visit with another provider would be for you? He/she explored this: | 1-Completely  2-Mostly  3-A little  4-Not at all |
| 12-To what extent did you and the doctor discuss your respective roles? (In other words, who is responsible for making decisions and who is responsible for what aspects of care)? | 1-Completely  2-Mostly  3-A little  4-Not at all |
| 13-To what extent did the doctor encourage you to the take the role you wanted in your own care? | 1-Completely  2-Mostly  3-A little  4-Not at all |
| Patient perception that doctor understands you as a whole person | |
| 14-How much would you say that this doctor cares about you as a person? | 1-Very much  2-Fair amount  3-A little  4-Not at all |

*Instructions at baseline visit: "Please answer the following questions thinking about your last visit with your kidney doctor." Instructions at 1-month follow-up visit: "Please answer the following questions thinking about the visit with your kidney doctor where you discussed the information on your personalized functioning report. If you did not discuss the report during a visit with your doctor, please answer with respect to your last visit with him/her."

**Total score = average of all scores; subscores =average of scores within subdomains. Scale, 1-4; score of 1= most patient-centered; score of 4=least patient-centered.

**Table S2.** Patient survey regarding the utility of the individualized functioning report.

| **Item** | **Possible responses** |
| --- | --- |
| Did anyone on your dialysis care team discuss the physical functioning report you received with you? | Yes/no |
| For patients answering “no” to first item: |  |
| Did you bring up your report in any of your visits? | Yes/no |
| Did you want to discuss the report you received with any of  your dialysis providers? | Yes/no |
| Did you discuss your report with anyone else? | Yes/no |
| (If “yes”) With whom did you discuss your report (check all  that apply)? | Primary care provider, Another non-dialysis provider, Spouse, Child, Other relative, Friend, Spiritual or religious advisor, Other |
| Do you plan to discuss the report with your provider in a future  visit? | Yes/no |
| Please tell us what would help you with this discussion. | Open response |
| Please tell us if there is anything we could change about the  report that would make you want to discuss the report with your  provider. | Open response |
| For patients answering “yes” to first item: |  |
| Who discussed the report with you (check all that apply)? | Kidney Doctor, Nurse, Social Worker, Dietitian, Other Dialysis Provider |
| Who brought up the report (check all that apply)? | Myself, Doctor, Nurse, Social Worker, Dietitian, Other |
| Did the doctor or anyone on your dialysis care team make any  recommendations or changes to your treatment plan after  discussing physical functioning? | Yes/no |
| What recommendations or changes to your care plan did they  suggest? (list) | Open response |
| Did you feel comfortable discussing your physical functioning  with your dialysis providers? | Yes/no |
| Did the physical functioning report lead to better  communication with your doctor? | Yes/no |
| Did you discuss the report with anyone else not on the dialysis  care team (check all that apply)? | Primary care provider, Another healthcare provider outside the dialysis care team, Spouse, Child, Other relative, Friend, Spiritual or religious advisor, Other, I did not discuss the report with anyone else |
| All patients: |  |
| Is there anything else not included on the report would you have wanted your provider to know about your physical functioning? | Open response |
| If this report were offered as part of usual care, how often would you be willing to do physical performance tests (for example, walking speed test) and answer surveys to receive this individualized report? | Once a month, Every 3 months, Every 6 months, Once a year, Never, Other |

**Table S3.** Provider survey regarding the utility of the individualized functioning report.

| **Item** | **Possible responses** |
| --- | --- |
| What is your role at the dialysis facility? | Nephrologist, Nurse, Social Worker, Dietitian, Other |
| About how many of these patient reports [example shown] did you receive? | Integer |
| Did you discuss the physical functioning report(s) you received with your patient(s)? | Yes, always, Yes, with some of the patients, No |
| For providers answering “no” to third item: |  |
| For what reasons did you not discuss the report (check all that  apply)? | I did not feel it was appropriate, I did not feel I was qualified, I did not feel it was my role, There was never enough time, Information in report was not important for patient care, Information in report was not actionable, I forgot about the report, Other |
| Did you *view* the reports on your patients? | Frequently, Sometimes, Rarely, Never |
| How often did you make any recommendations or changes to  patients' treatment plans after *viewing* the physical functioning  report *without* discussing the report with the patient)? | Frequently, Sometimes, Rarely, Never |
| (If not “never”) What types of recommendations/changes did  you make (check all that apply)? | Physical therapy, Patient-driven exercise program, Occupational therapy/home assessment, Depression workup, Cognitive assessment, New social services (*e.g*., Meals on Wheels, MARTA Mobility), Family consult, None, Other |
| For providers answering “yes, always” or “yes, with some of the patients” to third item: | |
| Did you feel comfortable discussing functioning with your  patients? | Yes/no |
| Did you or patients bring up the report on physical functioning  more often? | I Brought It Up More Often, The Patient Brought It Up More Often, Both of Us Brought It Up Equally |
| How often did you make any recommendations or changes to  patients' treatment plans after discussing the physical  functioning report? | Frequently, Sometimes, Rarely, Never |
| How often did you make any recommendations or changes to  patients' treatment plans after *viewing* the physical functioning  report *without* discussing the report with the patient)? | Frequently, Sometimes, Rarely, Never |
| (If not “never” to previous two items) What types of  recommendations/changes did you make (check all that  apply) | Physical therapy, Patient-driven exercise program, Occupational therapy/home assessment, Depression workup, Cognitive assessment, New social services (*e.g.*, Meals on Wheels, MARTA Mobility), Family consult , None, Other |
| Did the functioning report lead to better communication with  your patients? | Open response |
| All providers: |  |
| What type(s) of training would be helpful for improving your comfort with the report? | In-person training, Web training, Role modeling of patient-provider discussion, None (no training needed), Other |
| Who on the dialysis care team should discuss the report (check all that apply)? | Nephrologist, Nurse, Social worker, Dietitian, Primary care provider, No one should discuss the report, Other |
| What else would you have wanted to know about patients' physical functioning that was not included on the report? | Open response |
| Do you remember receiving email notifications that included patient reports? | Yes/no |
| Do you remember receiving email notifications that included patient reports? | Yes/no |
| Did you attempt to view patient reports on the EMR? | Yes/no |
| (If “yes”) Were the reports available on the EMR? | Yes/no |
| Were you present at any in-person meetings where the investigators presented the study? | Yes/no |
| Did you view either of the webinars describing the study and report (links provided in emails sent to providers)? | Yes/no |
| Did you contact the investigators to ask questions about the study or report? | Yes/no |
| How much did physical performance testing (*e.g*., gait speed) interfere with clinic flow? | A Lot, A Little, Not at all, Don't know/did not observe |
| How much did physical functioning surveys (*e.g*., activities of daily living) interfere with clinic flow? | A Lot, A Little, Not at all, Don't know/did not observe |
| If physical functioning measurement became part of usual care, are there ways to minimize the burden of measurement on dialysis staff? | Open response |

**Table S4.** Detailed functioning data on 43 patients receiving hemodialysis participating in pilot study (2/18-8/18), overall and by age and sex.

| **Domain/measure** | **Overall*** | | **Age, years** | | | **Sex** | | |
| --- | --- | --- | --- | --- | --- | --- | --- | --- |
|  |  |  | **<60** | **≥60** | ***P**** | **Female** | **Male** | ***P***** |
| *N (%)* | *43* | | *25 (58.1)* | *18 (41.9)* | *---* | *21 (48.8)* | *22 (51.2)* | *---* |
| Physical performance | | | | | | | | |
| SPPB scores, mean (SD) | | | | | | | | |
| Total score | 7.0 (3.1) | | 8.0 (2.9) | 5.7 (2.9) | *0.01* | 6.7 (3.1) | 7.4 (3.1) | *0.5* |
| Balance score | 2.9 (1.3) | | 3.3 (1.3) | 2.3 (1.3) | *0.02* | 2.7 (0.3) | 3.0 (1.4) | *0.4* |
| Gait speed score | 3.0 (1.2) | | 3.3 (1.0) | 2.6 (1.2) | *0.06* | 3.0 (1.2) | 3.0 (1.1) | *0.8* |
| Chair stand score | 1.1 (1.1) | | 1.4 (1.2) | 0.7 (0.8) | *0.03* | 1.0 (1.1) | 1.3 (1.1) | *0.4* |
| Actual gait speed (m/s), mean (SD) | 0.8 (0.2) | | 0.9 (0.2) | 0.7 (0.2) | *0.007* | 0.8 (0.2) | 0.8 (0.2) | *0.7* |
| Perceived physical functioning | | | | | | | | |
| PF score, mean (SD) | 57.6 (27.5) | | 67.0 (25.7) | 44.4 (25.1) | *0.007* | 51.8 (28.3) | 63.6 (25.9) | *0.2* |
| Activities of daily living | | | | | | | | |
| Any difficulty with BADLs, *n* (%) | | | | | | | | |
| Bathing | 8 (18.6%) | | 3 (12.0%) | 5 (27.8%) | *0.2* | 5 (22.7%) | 3 (14.3%) | *0.7* |
| Dressing | 9 (20.9%) | | 5 (20.0%) | 4 (22.2%) | *>0.9* | 4 (18.2%) | 5 (23.8%) | *0.7* |
| Toileting | 3 (7.0%) | | 2 (8.0%) | 1 (5.6%) | *>0.9* | 3 (13.6%) | 0 (0.0%) | *0.2* |
| Transferring | 14 (32.6%) | | 5 (20.0%) | 9 (50.0%) | *0.05* | 9 (40.9%) | 5 (23.8%) | *0.3* |
| Feeding | 0 (0.0%) | | 0 (0.0%) | 0 (0.0%) | *---* | 0 (0.0%) | 0 (0.0%) | *---* |
| Continence | 11 (25.6%) | | 5 (20.0%) | 6 (33.3%) | *0.5* | 5 (22.7%) | 6 (28.6%) | *0.7* |
| Any difficulty with IADLs, *n* (%) | | | | | | | | |
| Telephone | | 0 (0.0%) | 0 (0.0%) | 0 (0.0%) | *---* | 0 (0.0%) | 0 (0.0%) | *---* |
| Shopping | | 11 (25.6%) | 5 (20.0%) | 6 (33.3%) | *0.5* | 7 (31.8%) | 4 (19.1%) | *0.5* |
| Food preparation | | 12 (27.9%) | 8 (32.0%) | 4 (22.2%) | *0.4* | 7 (31.8%) | 5 (23.8%) | *0.7* |
| Housework | | 18 (41.9%) | 8 (32.0%) | 10 (55.6%) | *0.2* | 10 (45.5%) | 8 (38.1%) | *0.8* |
| Laundry | | 12 (27.9%) | 6 (24.0%) | 6 (33.3%) | *0.5* | 5 (22.7%) | 7 (33.3%) | *0.5* |
| Transportation | | 14 (32.6%) | 7 (28.0%) | 7 (38.9%) | *0.5* | 9 (40.9%) | 5 (23.8%) | *0.3* |
| Medicine | | 4 (9.3%) | 2 (8.0%) | 2 (11.1%) | *>0.9* | 2 (9.1%) | 2 (9.5%) | *>0.9* |
| Finance | | 5 (11.6%) | 2 (8.0%) | 3 (16.7%) | *0.6* | 3 (13.6%) | 2 (9.5%) | *>0.9* |
| Falls | | | | | | | | |
| History of falls, *n* (%): | |  |  |  |  |  |  |  |
| Fell in past year | | 15 (34.9%) | 8 (32.0%) | 7 (38.9%) | *0.8* | 6 (27.3%) | 9 (42.9%) | *0.3* |
| 1 fall in past year | | 5 (11.6%) | 3 (12.0%) | 2 (11.1%) | *0.9* | 3 (13.6%) | 2 (9.5%) | *0.3* |
| 2 falls in past year | | 10 (23.3%) | 5 (20.0% | 5 (27.8%) |  | 3 (13.6%) | 7 (33.3%) |  |
| Injury fall in past year | | 1 (2.3%) | 1 (4.0%) | 0 (0.0%) | *>0.9* | 1 (4.6%) | 0 (0.0%) | *>0.9* |
| Reasons for falls (among *n*=15 fallers): | | | | | | | | |
| Dizziness | | 6 (40.0%) | 4 (50.0%) | 2 (28.6%) | *0.6* | 2 (33.3%) | 4 (44.4%) | *>0.9* |
| Fainted | | 4 (26.7%) | 3 (37.5%) | 1 (14.3%) | *0.6* | 1 (16.7%) | 3 (33.3%) | *0.6* |
| Lightheadedness | | 4 (26.7%) | 3 (37.5%) | 1 (14.3%) | *0.6* | 1 (16.7%) | 3 (33.3%) | *0.6* |
| Heart racing | | 2 (13.3%) | 2 (25.0%) | 0 (0.0%) | *0.5* | 0 (0.0%) | 2 (22.2%) | *0.5* |
| Confusion | | 1 (6.7%) | 1 (12.5%) | 0 (0.0%) | *>0.9* | 0 (0.0%) | 1 (11.1%) | *>0.9* |
| Pain | | 1 (6.7%) | 1 (12.5%) | 0 (0.0%) | *>0.9* | 0 (0.0%) | 1 (11.1%) | *>0.9* |
| Leg weakness | | 8 (53.3%) | 4 (50.0%) | 4 (57.1%) | *>0.9* | 4 (66.7%) | 4 (44.4%) | *0.6* |
| Weakness | | 8 (53.3%) | 4 (50.0%) | 4 (57.1%) | *>0.9* | 3 (50.0%) | 5 (55.6%) | *>0.9* |
| Unstable joint | | 7 (46.7%) | 2 (25.0%) | 5 (71.4%) | *0.1* | 5 (83.3%) | 2 (22.2%) | *0.04* |
| Lost balance | | 10 (66.7%) | 6 (75.0%) | 4 (57.1%) | *0.6* | 5 (66.7%) | 6 (66.7%) | *>0.9* |
| Rushing | | 4 (26.7%) | 1 (12.5%) | 3 (42.9%) | *0.3* | 2 (33.3%) | 2 (22.2%) | *>0.9* |
| Tripped | | 7 (46.7%) | 3 (37.5%) | 4 (57.1%) | *0.6* | 3 (50.0%) | 4 (44.4%) | *>0.9* |
| Environment | | 5 (33.3%) | 2 (25.0%) | 3 (42.9%) | *0.6* | 4 (66.7%) | 1 (11.1%) | *0.09* |
| Low lighting | | 2 (13.3%) | 1 (12.5%) | 1 (14.3%) | *>0.9* | 1 (16.7%) | 1 (11.1%) | *>0.9* |
| Footwear | | 1 (6.7%) | 0 (0.0%) | 1 (14.3%) | *0.5* | 1 (16.7%) | 0 (0.0%) | *0.4* |
| Other | | 4 (26.7%) | 2 (25.0%) | 2 (28.6%) | *>0.9* | 1 (16.7%) | 3 (33.3%) | *0.6* |
| FES score, mean (SD) | | 18.4 (14.8) | 16.8 (14.3) | 20.7 (15.6) | *0.4* | 19.8 (18.3) | 17.0 (10.2) | *0.5* |
| Fear of falling, *n* (%) | | 2 (4.7%) | 1 (4.0%) | 1 (5.6%) | *>0.9* | 2 (9.1%) | 0 (0.0%) | *0.5* |
| Community mobility | | | | | | | | |
| LSA score, mean (SD) | | 55.2 (26.6) | 65.0 (54.5) | 41.6 (22.6) | *0.003* | 49.6 (27.9) | 61.1 (24.5) | *0.2* |
| Not visiting neighborhood without personal assistance, *n* (%) | | 9 (20.9%) | 3 (12.0%) | 6 (33.3%) | *0.1* | 7 (31.8%) | 2 (9.5%) | *0.1* |

BADL, basic activity of daily living; ESRD, end-stage renal disease; IADL, instrumental activity of daily living; IQR, interquartile range; LSA, Life-Space Assessment (scale, 0-120; higher scores = greater community mobility); FES, Falls Efficacy Scale (scale 0-100; higher scores = greater fear of falling during daily tasks; scores of ≥70 = fear of falling); PF, physical functioning subscale (scale 0-100; higher scores = better perceived functioning); PPPC, Patient Perception of Patient-Centeredness (scale, 1-4; lower scores = more patient-centered); SPPB, Short Physical Performance Battery (scale, 0-12; higher scores=better performance).

**N*=43 for all except gait speed [*N*=41 (n=2 did not compete walking course)] and causes of falls (among *n*=15 fallers only).

**By *t* or Fisher’s exact test, as appropriate.

**Table S5.** Pairwise correlations* between physical functioning scores among 43 patients receiving hemodialysis participating in pilot study, 2/18-8/18.

|  | **Physical performance** | **Perceived functioning** | **BADLs** | **IADLs** | **Falls** | **Life-space** |
| --- | --- | --- | --- | --- | --- | --- |
| **Physical performance** | 1.00 |  |  |  |  |  |
| **Perceived functioning** | 0.71  *<0.001* | 1.00 |  |  |  |  |
| **BADLs** | 0.50  *<0.001* | 0.65  *<0.001* | 1.00 |  |  |  |
| **IADLs** | 0.46  *0.002* | 0.66  *<0.001* | 0.77  *<0.001* | 1.00 |  |  |
| **Falls** | 0.29  *0.06* | 0.19  *0.2* | 0.32  *0.04* | 0.34  *0.03* | 1.00 |  |
| **Life-space** | 0.56  *<0.001* | 0.69  *<0.001* | 0.47  *0.002* | 0.40  *0.008* | 0.16  *0.3* | 1.00 |

BADL, basic activity of daily living; IADL, instrumental activity of daily living.

Physical performance = Short Physical Performance Battery score (scale 0-12; higher scores = better performance); perceived functioning = physical functioning subscale on Kidney Disease Quality of Life questionnaire (scale 0-100; higher scores = higher perceived functioning) ; BADLs = count of BADLs with any impairment (scale 0-6); IADLs = count of IADLs with any impairment (scale 0-8); falls = number of patient-reported falls in prior year (range 0-2); life-space = Life-Space Assessment score (scale 0-120; higher scores = greater community mobility).

*All correlation coefficients represent absolute values.

**Box S1.** Example initial and reminder emails sent to providers in the pilot study.

**Example provider email (after assessment; with report attached):**

Dear Dr. X:

Attached is the physical functioning report generated for one of your patients, *Y*, who is participating in our INFORMED study. The patient also received a printout of this report to take home and review on the day of assessment. Please review the report and discuss it with your patient over the next month. This discussion can be one-on-one or with the care team. You can also spread the discussion over multiple encounters if needed to address concerns you or the patient has. Note that the report will also be uploaded onto the EMR so it will be available chair-side. To view functioning report: search for patient, navigate to Medical Records, Patient Charts, and Imported Documents.

If you have any questions about the study or the report, please consult the recorded webinars describing the [study](https://youtu.be/1ivHMgMuoOI) and the [report](https://youtu.be/n8cR-dOaNUM) and/or the information our study [website](http://medicine.emory.edu/geriatrics-gerontology/labs/plantinga-lab/index.html), which also includes links to the webinars. Please feel free to contact the study PI (Dr. [Laura Plantinga](mailto:laura.plantinga@emory.edu)) with any questions as well.

Thank you for your participation!

The INFORMED Study Team

**Example reminder email (sent in the week before interdisciplinary rounds):**

Dear Dialysis Care Providers:

This a monthly reminder to discuss the physical functioning report(s) you have received with your patients who are participating in INFORMED study. These reports have been e-mailed to you as they were generated and are also available on the EMR, so you can refer to them chair-side. If you would like another copy of a patient’s report please let us know.

If you have any questions about the study or the report, please consult the recorded webinars describing the [study](https://youtu.be/1ivHMgMuoOI) and the [report](https://youtu.be/n8cR-dOaNUM) and/or the information our study [website](http://medicine.emory.edu/geriatrics-gerontology/labs/plantinga-lab/index.html), which also includes links to the webinars. Please feel free to contact the study PI (Dr. [Laura Plantinga](mailto:laura.plantinga@emory.edu)) with any questions as well.

Thank you for your participation!

The INFORMED Study Team

**Figure S1.** Initial physical functioning report on a hypothetical patient receiving hemodialysis, which was used in focus groups for feedback; key modifications based on qualitative feedback from focus groups; and final version of the report that was used for testing in the pilot.

**
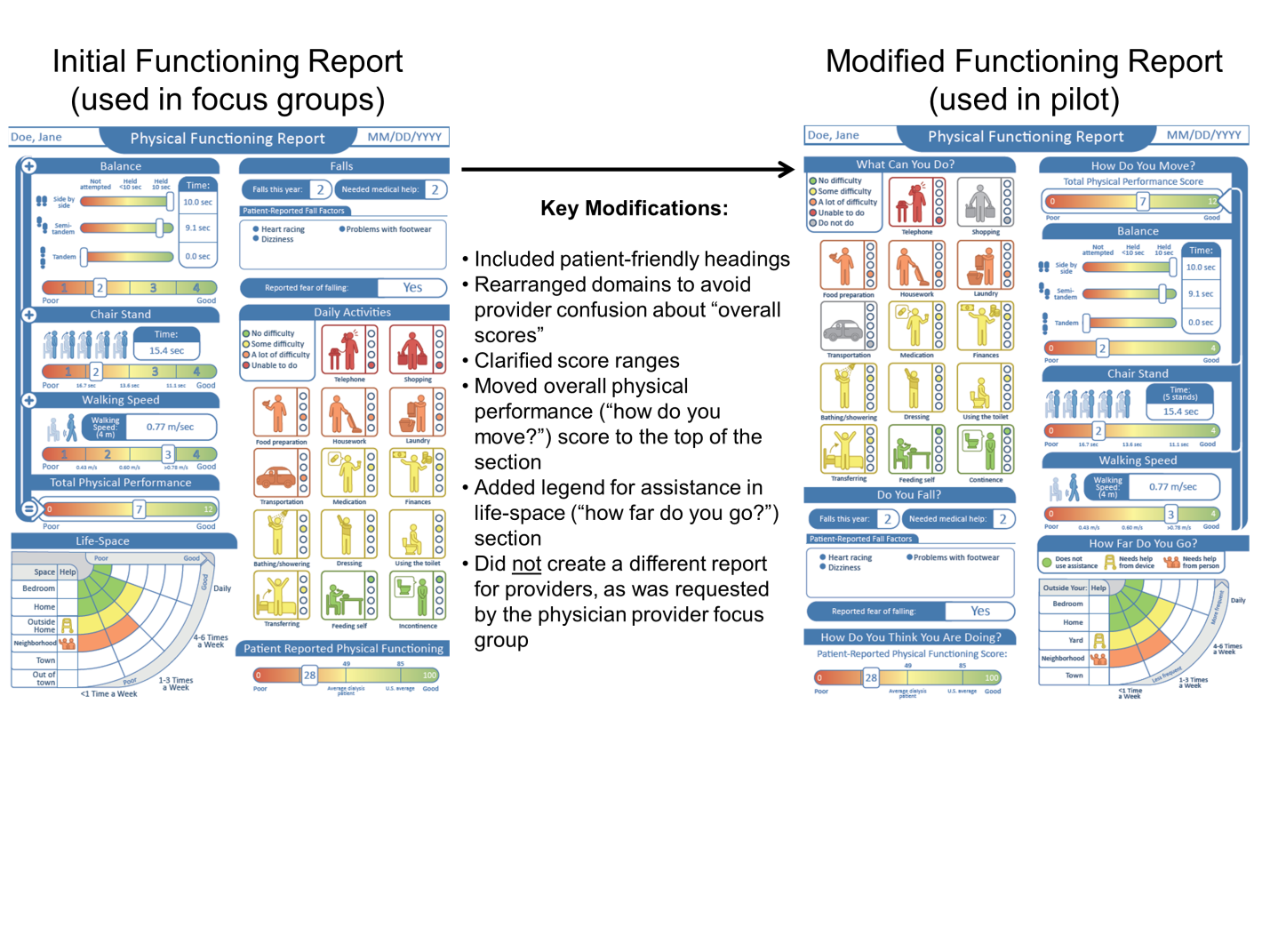
**

**Figure S2.** Participant flow in pilot study. *All participants who completed a baseline visit also completed the 1-month follow-up visit.

**
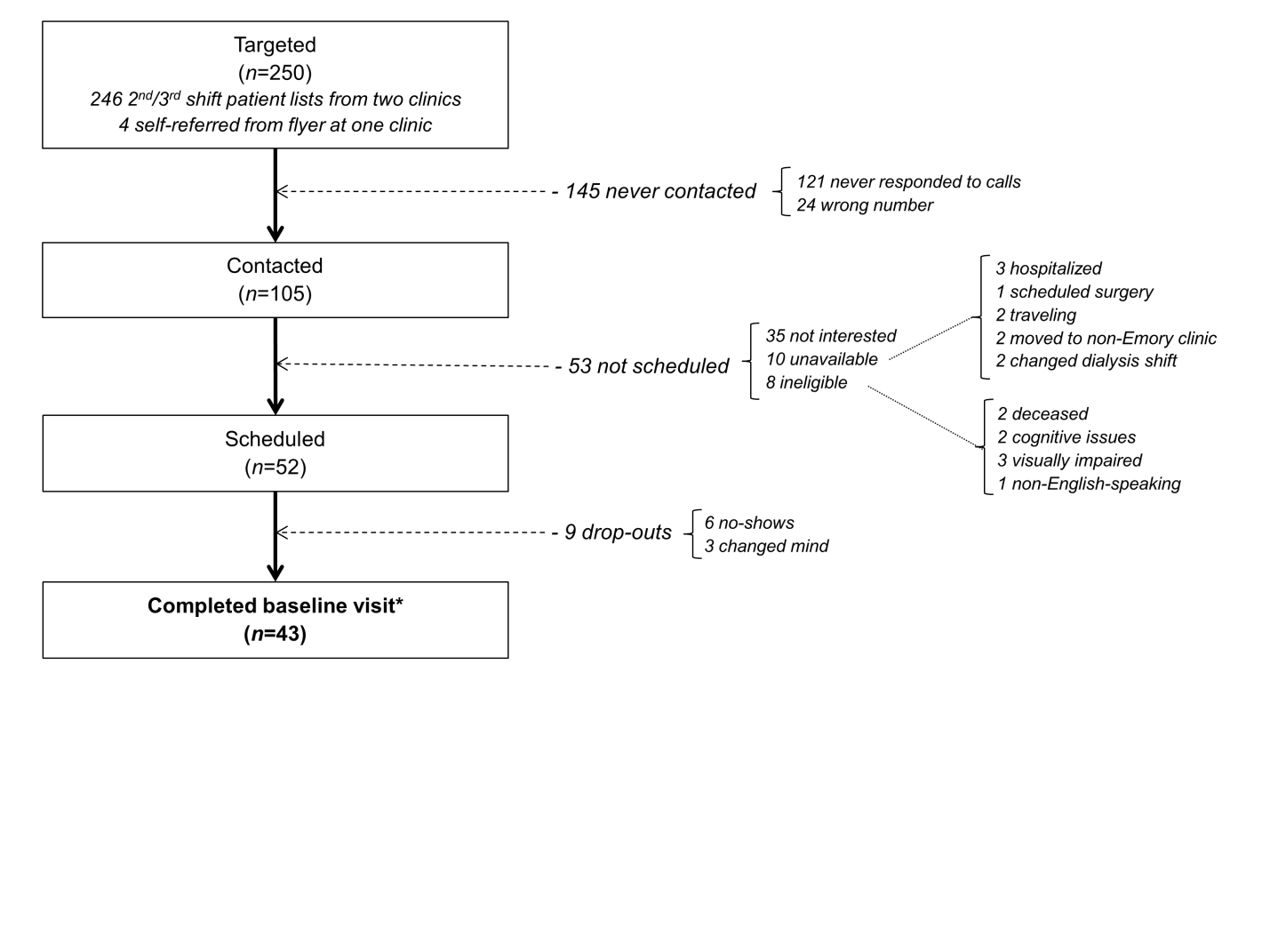
**

**Figure S3.** Overlap of limitations in physical functioning domains among 32 patients receiving hemodialysis participating in the pilot study, 2/18-8/18, with ≥1 limitation [11/43 (25.6%) participants had no impairments]. **A**, UpSet ^29^ visualization and counts of all intersecting sets of impairments; **B**, Euler ^30^ diagram of intersecting sets of limitations.

*Limitation definitions: BADL/IADL, any difficulty (vs. “no difficulty”); falls, any fall in past year or fear of falling (Falls Efficacy Score ≥ 70); physical performance, total Short Physical Performance Battery score ≤6 (vs. >6), or missing (did not do test); perceived functioning, total physical functioning score <50 (vs. >=50); and life-space, inability to get to neighborhood level without personal assistance.*

**
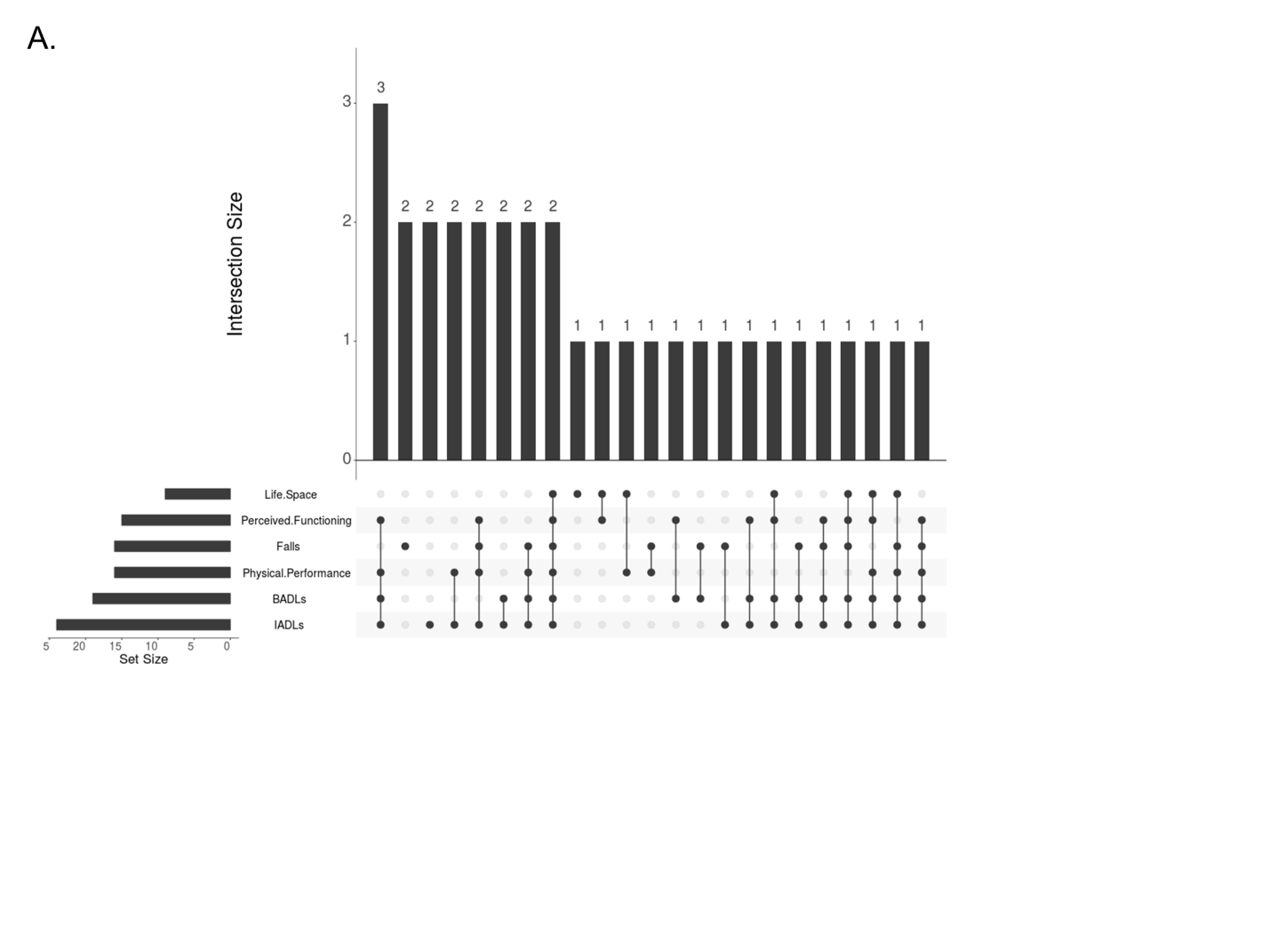

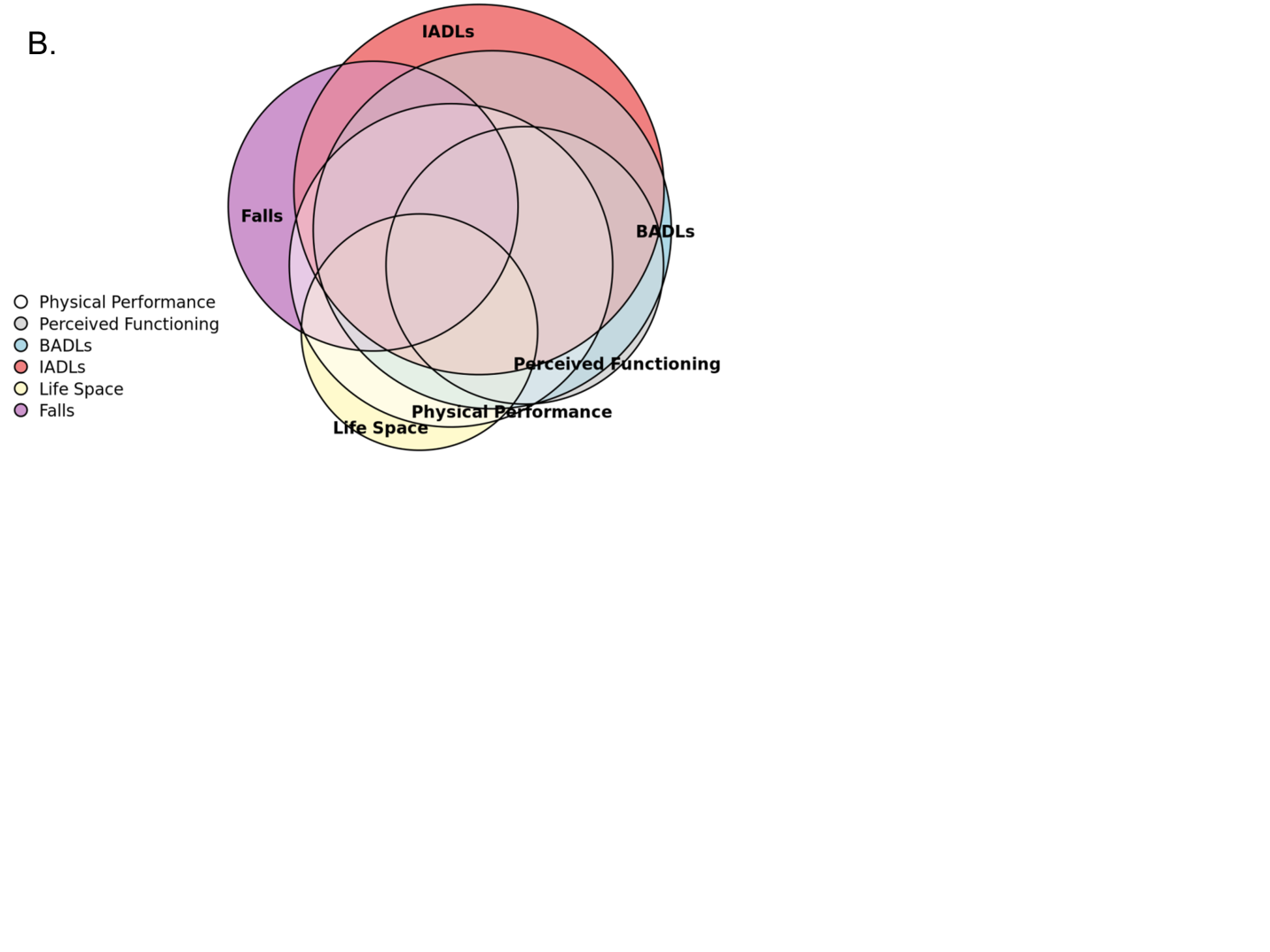
**
